# Alkane biosynthesis gene expression and its increased production in recombinant cyanobacteria

**DOI:** 10.1002/2211-5463.70009

**Published:** 2025-03-06

**Authors:** Misato Nagao, Takato Ozaki, Hirofumi Fukuda, Yu Kanesaki, Munehiko Asayama

**Affiliations:** ^1^ College of Agriculture Ibaraki University Ibaraki Japan; ^2^ United Graduate School of Agricultural Science Tokyo University of Agriculture and Technology Tokyo Japan; ^3^ Research Institute of Green Science and Technology Shizuoka University Shizuoka Japan

**Keywords:** alkane, cyanobacteria, hydrocarbon fuel, primer extension, promoter, transcription

## Abstract

Microalgae such as cyanobacteria convert CO_2_ to compatible drop‐in fuels, such as alkanes. However, the production yield is approximately 0.05–1.0% of the dry weight of natural algae. Here, we aimed to study the role of transcriptional expression, mRNA molecular structure and culture‐dependent accumulation of alkanes from two cyanobacteria species. The transcription start sites of the alkane biosynthesis genes *ado* and *aar* were identified in the representative cyanobacteria strains *Synechocystis* sp. PCC 6803 and *Limnothrix* sp. SK1‐2‐1, which produce heptadecane and pentadecane, respectively. This characterisation revealed the potential promoters and unique mRNA structures of the *ado* and *aar* genes in these species. Transcripts from these genes were induced more in the nitrogen‐depleted BG11 (BG11‐N) culture than in the BG11 culture, although the biomass was reduced, and as such the amount of alkanes obtained per unit medium was greater for BG11 than for BG11‐N. PCC 6803 transconjugants carrying alkane biosynthesis genes from PCC 6803 or SK1‐2‐1 showed an approximately 1.8‐ to 2.3‐fold increase in heptadecane production compared to the control strain when grown on BG11 cultures without any nitrogen depletion. These results suggest that not only the enzymes ADO/AAR but also the intracellular production of fatty acyl‐ACP substrates may be important for the mass production of target alkanes.

## Graphical abstract

The transcription and mRNA structures of the alkane biosynthesis genes *ado*/*aar* were analysed in the representative cyanobacteria PCC 6803 and SK1‐2‐1, which produce C_17_H_36_ and C_15_H_32_, respectively. PCC 6803 transconjugants carrying these genes showed a 1.8‐ to 2.3‐fold increase in C_17_H_36_ production. The results indicate that ADO/AAR activities, together with fatty acyl‐ACP mass, are important to produce target alkanes.

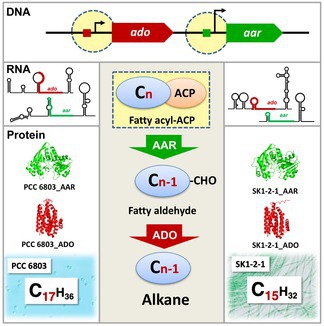

**Abbreviations**
AAR: acyl‐ACP reductase
ADO: aldehyde‐deformylating oxygenase
GC–MS: gas chromatography–mass spectroscopy
GT: glucose tolerance
S&L: stem and loop
TAG: triacylglycerol
TC: transconjugant
TEM: transmission electron microscopy

Microalgae produce organic materials that are beneficial to humans through carbon‐neutral and energy‐saving bioprocesses. Although microalgae biorefinery holds prospect to help solve the challenges faced by humanity, there is a need for comprehensive approach to the production process (supply chain) at the genetic, culture, recovery, production, processing, quality control and distribution levels. One such method involves the production of biofuels from photosynthetic microbial systems. Cyanobacteria convert CO_2_ to compatible drop‐in fuels, such as alkanes, which are straight‐chain hydrocarbons and prominent components of petrol that are derived from the decay of biological debris over millions of years [1]. Alkanes are produced in nature by bacteria, archaea, insects, plants and potentially fungi [2, 3, 4, 5, 6]. Cyanobacteria produce C15 or C17 alkanes synthesised from precursors in fatty acid metabolism, although they account for only a portion of fatty acid materials [7, 8] (Fig. 1). Two cyanobacterial biosynthetic pathways of alkanes and alkenes have been identified. One pathway involves the reduction/deformylation of the corresponding fatty acyl‐acyl carrier proteins (ACPs), precursors of alkanes and free fatty acids, by a set of soluble acyl‐ACP reductase (AAR)/aldehyde‐deformylating oxygenase (ADO) enzymes, respectively, resulting in the production of alkanes or alkenes (AAR/ADO type) [9, 10]. The other pathway involves elongation and decarboxylation of the corresponding fatty acids by a polyketide synthase (PKS)‐like complex, producing terminal alkenes (PKS type) [11]. These two metabolic pathways for the biosynthesis of alka(e)nes have not been previously identified in the same cyanobacterial strain.

**Fig. 1:**
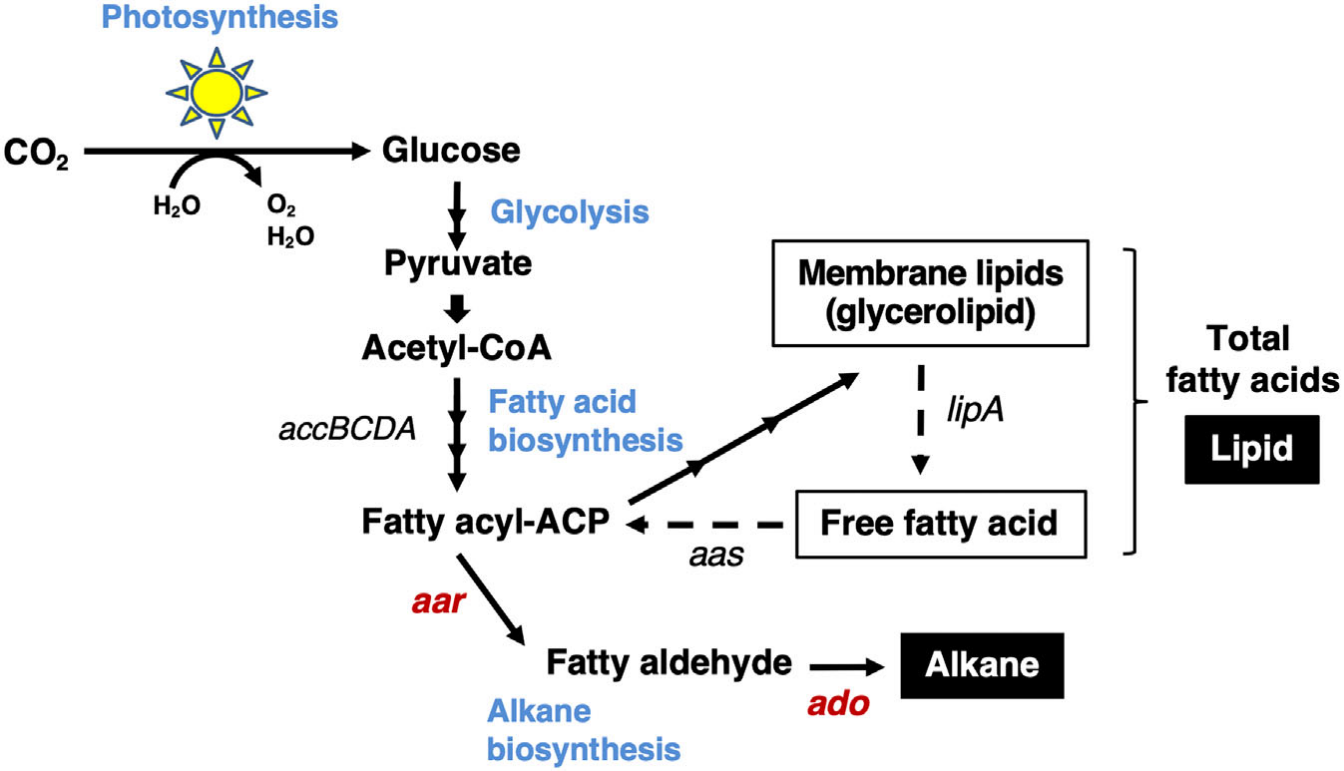
Schematic overview of alkane and lipid biosynthesis in cyanobacteria. Genes encoding key enzymes for this biosynthesis are *accBCDA* (multi‐subunit acetyl‐CoA carboxylase), *lipA* (lipase), *aas* (acyl‐acyl carrier protein synthetase), *aar* (fatty acyl‐ACP reductase) and *ado* (fatty aldehyde‐deformylating oxygenase).

The principal role of alkanes in cyanobacteria was disclosed by Berla et al. [12], who reported that C17 alkanes (medium‐chain hydrocarbons) regulate the redox balance and reductant partitioning in the unicellular cyanobacterium *Synechocystis* sp. Pasteur Culture Collection (PCC) 6803 under cold stress condition. PCC 6803 strain harbours a set of alkane synthesis genes (*sll0209* for AAR/*sll0208* for ADO type) that produce heptadecane (C_17_H_36_), which is useful as light oil. PCC 6803 cyanobacterium is the first photosynthetic organism to have its complete genome sequence revealed and a well‐known model that is easily amenable to genetic engineering [13]. In previous studies, improvement in alkane production in PCC 6803 strain was achieved through genetic engineering under continuous light conditions [10, 14, 15]. Reducing the cost of biofuel production during the cell growth and alkane production phases is indispensable. In the same group of PCC 6803 strains, the PCC 6803GT (glucose‐tolerant) strain possesses a transposase at the *sll1474* loci and some flanking regions are missing in the mutant [16] which can grow mixotrophically under continuous light conditions and heterotrophically under dark conditions with a short daily pulse of dim light [17]. Cyanobacteria usually produce heptadecane equivalent to diesel fuel, and some strains can produce pentadecane (C_15_H_32_) equivalent to jet fuel; however, their production is approximately 0.05–1.0% of the dry weight of natural algae, depending on the medium and culture conditions. The amount of production depends significantly on the culture medium and conditions [18].

Nutrient limitations, particularly nitrogen limitation, have been suggested to have a significant impact on lipid accumulation in microalgal cells, as demonstrated by the dominant proportion of accumulated triacylglycerol (TAG) [19]. Additionally, nitrogenous and sulphurous limitations may enhance TAG production, which has been shown to reach 30% to 75% in *Chlorella* and *Parachlorella* species, respectively [20, 21, 22, 23, 24, 25, 26, 27, 28]. Furthermore, *Chlamydomonas reinhardtii*, engineered using a phosphoric elimination‐inducible promoter for overexpression of cloned genes, showed enhanced TAG accumulation during cell growth under phosphorus‐depleted conditions [29]. These findings demonstrate that the depletion of nutrient microelements in a medium is an easy and effective method for increasing the lipid content in cells. However, this may not be appropriate because cell growth inhibition and total biomass reduction affect various aspects of metabolism in cyanobacteria. We previously succeeded in enhancing the accumulation of heptadecane in PCC 6803KZ (originated from the Kazusa DNA Research Institute as a wild‐type (WT) glucose‐intolerant strain) transconjugated with a cyanobacterial expression vector carrying *aar*/*ado* genes, and the strain was cultivated under nitrogen‐depleted conditions with continuous light (Table 1) [31, 33].

**Table 1:** Plasmids and strains used in this study.

Plasmid and strain	Type	Description	Source or reference
Plasmid			
pAM461c	Expression vector	pVZ321 + P*psbA2*_ΔAT, Cm^R^	Asayama [30]
pTM‐ALK11	Expression vector	pAM461c + PCC 6803 a*do* & *aar* genes, Cm^R^	Yoshida et al. [31]
pSK‐ALK461c	Expression vector	pAM461c + SK1‐2‐1 a*do* & *aar* genes, Cm^R^	Maruta [32]
R751	Helper plasmid	IncP, Tra^+^, Tp^R^	Meyer and Shapiro [33]
psbA2‐4	Cloned plasmid	PCC 6803 *psbA2* + upstream region in pUC119B, Amp^R^	Imamura et al. [34]
pUC6803sll0208up	Cloned plasmid	pUC119BNXN + PCC 6803 *ado* upstream region from −177 to +84, Amp^R^	This article
pUC6803sll0209up	Cloned plasmid	pUC119BNXN + PCC 6803 *aar* upstream region from −173 to +55, Amp^R^	This article
pUCSKsll0208up	Cloned plasmid	pUC119BNXN + SK1‐2‐1 *ado* upstream region from −162 to +76, Amp^R^	This article
pUCSKsll0209up	Cloned plasmid	pUC119BNXN + SK1‐2‐1 *aar* upstream region from −137 to +52, Amp^R^	This article
*Escherichia coli*			
DH5αMCR	Host cell	λ‐, F‐, *sup*E44, *endA1*, *recA1*, *gyrA96*, *thi*, *hsdR17*, Δ(*lacZYA*‐*argF*) U169, φ80d*lacZ*Δ*M15*, *relA1*, *mcrA*, Δ(*mcrBC*, *mrr*, *hsdRMS*), *deoR*	Cosmo Bio Co. Ltd.
Cyanobacteria			
*Limnothrix* sp. SK1‐2‐1	Wild‐type	Wild‐type strain producing C15 alkane (C_15_H_32_), phycocyanin, and extracellular polysaccharide	Sugawara et al. [18] Aoki et al. [35, 36]
*Synechocystis* sp. PCC 6803GT	Glucose tolerance	PCC 6803GT producing C17 alkane (C_17_H_36_)	Pasteur Culture Collection (Paris, France)
*Synechocystis* sp. PCC 6803GT_pAM461c	Transconjugant	PCC 6803GT transconjugant harbouring pAM461c, Cm^R^	Yoshida et al. [31]
*Synechocystis* sp. PCC 6803GT_pTM‐ALK11	Transconjugant	PCC 6803GT transconjugant harbouring pTM‐ALK11, Cm^R^	Yoshida et al. [31]
*Synechocystis* sp. PCC 6803GT_pSK‐ALK461c	Transconjugant	PCC 6803GT transconjugant harbouring pSK‐ALK461c, Cm^R^	Maruta [32]

Amp^R^, Ampicillin resistance; Cm^R^, chloramphenicol required for *E*. *coli* (at concentration of 25 μg·mL^−1^) or PCC 6803 transconjugants (at concentration of 12.5 μg·mL^−1^); PpsbA, *psbA* promoter; Tp^R^, trimethoprim resistance.

In this study, we have performed a series of analyses of the alkane biosynthesis genes *ado* and *aar* with respect to transcriptional expression, mRNA molecular structure, and culture‐dependent accumulation of alkanes in *Synechocystis* sp. PCC 6803 and *Limnothrix* sp. SK1‐2‐1, which produce C17‐ or C15‐alkanes, respectively [18, 35, 36]. In addition to confirming an increase in alkane production by the recombinant strain PCC 6803GT, an intracellular state was also observed.

Based on the new knowledge obtained through the above studies, strategies for the efficient production of alkanes with the desired carbon chain length in cyanobacteria are discussed.

## Materials and methods

### Plasmids, bacterial strains, nucleotide sequences

The plasmids and bacterial strains used in this study are listed in Table 1. The accession numbers for the PCC 6803 genome sequence involving *ado* (*sll0208*)/*aar* (*sll0209*) and the nucleotide sequence involving the SK1‐2‐1 *ado*/*aar* region are BA000022 and LC815305, respectively.

### Cultivation conditions for preparing total RNAs used in primer extension

**Fig. 2:**
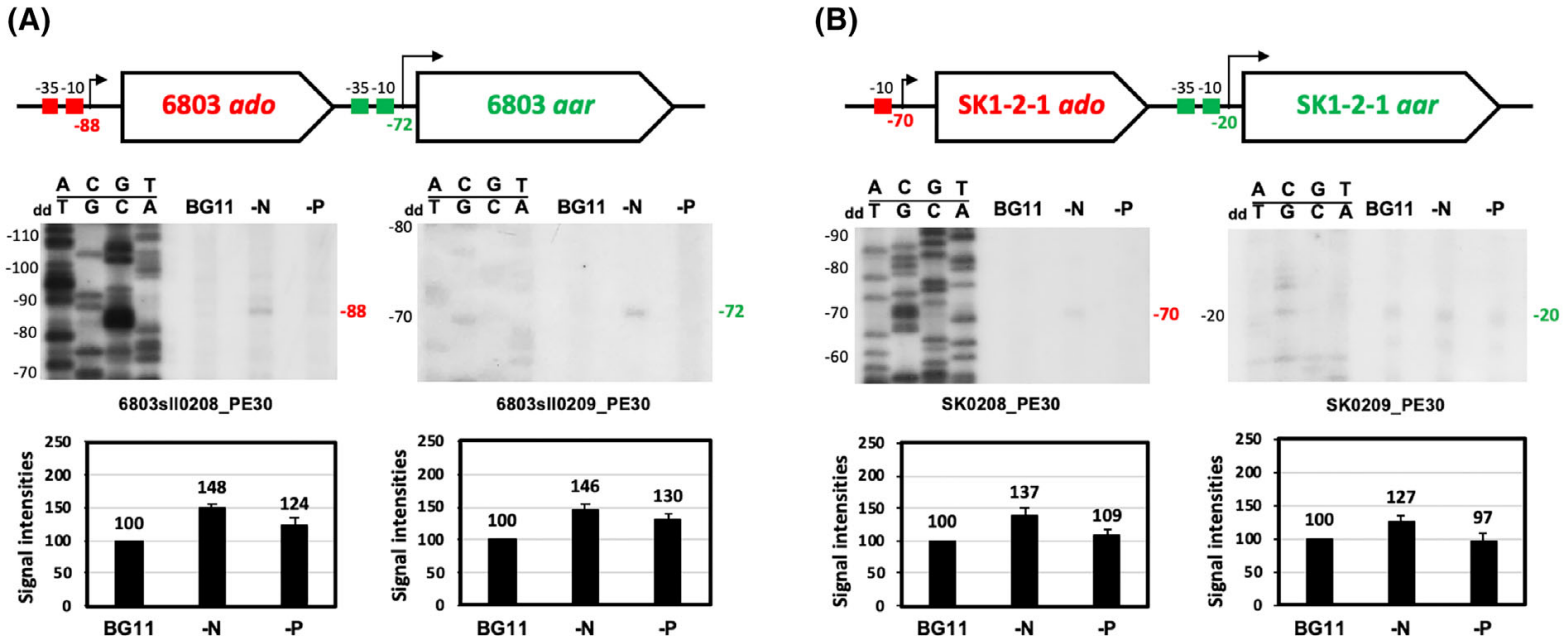
Gene expression and mRNA accumulation of alkane biosynthesis genes in cyanobacteria. The structures of *ado*/*aar* genes in *Synechocystis* sp. PCC 6803 (A) or *Limnothrix* sp. SK1‐2‐1 (B) is shown at the top of each panel. The results of primer extension analysis with the specific primers (‘X’_PE30) and the signal corresponding to transcription start site (TSS) are indicated at the medium of respective panels. Signal intensities as mRNA accumulations from TSS on X‐ray films were measured and also shown at the bottom in relative values as % under cultivation condition (BG11, ‐N, ‐P) for 1 day (Materials and Methods, Fig. S1). Error bars represent the standard error (6803 *ado*, *n* = 3; 6803 *aar*, *n* = 2; SK1‐2‐1 *ado*, *n* = 2; SK1‐2‐1 *aar*, *n* = 3). Sequence ladders are shown along with ACGT of the top strand and dideoxy (dd) TGCA of the bottom (^32^P‐labelled primer) strand.

In routine transplantation cultures, *Synechocystis* sp. PCC 6803 and *Limnothrix* sp. SK1‐2‐1 cells were cultivated in 250 mL of BG11 medium [37] in 1‐L flasks. In the main culture experiment, a 75‐mL aliquot of cell culture was harvested by centrifugation, and the cell pellet was suspended in 50 mL of BG11, BG11 without NaNO_3_ (nitrogen depletion, BG11‐N, BG11_0_) [37], BG11 without K_2_HPO_4_·3H_2_O (phosphorus depletion, BG11‐P), or BG11 without MgSO_4_·7H_2_O (sulphur depletion, BG11‐S) medium in a 200‐mL Erlenmeyer flask. The flask was kept in a 2% CO_2_ incubator (CF‐415; TOMY, Tokyo, Japan) at 30 °C. The culture was reciprocally shaken at 40 rpm under white light irradiation of 30 μmol·photons·m^−2^·s^−1^ for one day (Fig. 2; Fig. S1A). After incubation, the cells were harvested by centrifugation and total RNAs were extracted (Fig. S1B) and they were subjected to primer extension analysis for 5′‐end mapping of transcripts [38, 39]. In this analysis, the PCR‐synthesised (linear form) or recombinant plasmid (supercoil form) DNAs (Fig. S1C) were used as templates for sequencing the ladders.

### Cultivation conditions for cyanobacterial transconjugants

In routine transplantation cultures, PCC 6803_WT cyanobacteria were grown in BG11 liquid medium [40], and TCs harbouring pAM461c, pTM‐ALK11 or pSK‐ALK461c were grown in BG11 medium containing chloramphenicol at a final concentration of 12.5 μg·mL^−1^. In the main culture experiment, a 30‐mL aliquot of cell culture from 5‐L of tank‐cultured cyanobacteria was harvested by centrifugation, and the cell pellet was suspended in 50 mL of BG11, BG11 without NaNO_3_ (nitrogen depletion, ‐N) [40], BG11 without K_2_HPO_4_·3H_2_O (phosphorus depletion, ‐P) or BG11 without MgSO_4_·7H_2_O (sulphur depletion, ‐S) medium in a 200‐mL Erlenmeyer flask. The flask was maintained at 2% (Fig. 4; Table S2, Fig. S4) or 0.04% (Figs 5 and 6; Table S3, Figs S3 and S5) CO_2_ incubator at 30 °C. The culture was reciprocally shaken at 40 rpm under white light irradiation of 30 μmol·photons·m^−2^·s^−1^ for 6 days (Figs 4 and 6; Table S2, Figs S3 and S4) or 4 weeks (Fig. 5; Table S3, Fig. S4), depending on the situation. After incubation, the cells were harvested by centrifugation and subjected to alkane measurement or microscopic observation.

### Preparation of total DNA and RNA

Total DNA was prepared from 50‐mL cell culture using a previously described method [41, 42] and subjected to PCR. Total RNA was prepared from 50‐mL of cell culture using a previously described method [30, 43] and subjected to primer extension analysis (Fig. S1).

### 5′‐end mapping of transcripts

5′‐end mapping of *ado*/*aar* transcripts (Fig. 2; Fig. S1) was performed by the primer extension method [41, 42, 43] as follows. Total RNA (1–10 μg) and ^32^P‐labelled specific primers (6803sll0208_PE30, 5′‐GACAATGGCGTTGATGCGGCTATAGGCGTC‐3′; 6803sll0209_PE30, 5′‐CAGCAACCGCTTGGGCGTGTTCTAAACTCG‐3′; SK0208_PE30, 5′‐CATTGATGCGGCTGTAGGCATCTTTGTAGG‐3′; SK0209_PE30, 5′‐CGATTTCGTGCGTTCTTTCTAAGCTGGGGG‐3′) were mixed and annealed (Fig. 2; Fig. S1). After this, cDNA was synthesised by reverse transcriptase ReverTra Ace (TRT‐101; TOYOBO Co. Ltd., Osaka, Japan) at 42 °C. The resulting gel was electrophoresed on 6% denatured polyacrylamide gel containing 8 m urea. Subsequently, the gel was subjected to autoradiography using an X‐ray film for 13–20 days, depending on the band signal intensities. The band signal intensities corresponding to the number of transcripts were verified using BIO1D in the MEGA BIO‐PRINT‐1100 software (Vilber Lourmat, Marne La Vallée, France) and are shown in relative values as percentage [38]. For sequencing ladder, 2.5‐kb DNA fragments of *ado*/*aar* region were amplified by PCR with a set of primers (6803sll0208_FwUP500Sm+, 5′‐TCCCCCGGGGAACATAGTGTCGTGCTCCAAGGAGCTAGC‐3′ and 6803sll0209_Rv30Bm, 5′‐CGGGATCCACGCCCTATCCTGTCGGCCTAAAGAGCTAC‐3′, or SK0208_FwUP500Sm, 5′‐TCCCCCGGGGAATCAAGACCAATTTCGCTGAAATTCAGG‐3′ and SK0209_Rv30Bg, 5′‐GAAGATCTAAGCTGGCGCATGGGGTCTTTAGCCGACAG‐3′), respectively, and genomic DNA of PCC 6803 or SK1‐2‐1 as templates. These PCR fragments were sequenced using the dideoxy method with the same ^32^P‐labelled specific primers in the respective primer extension reactions. A sequence ladder of *Synechocystis* sp. PCC 6803 *psbA2* was used as an internal control in which plasmid psbA2‐4 and ^32^P‐labelled primer PsbA2.3‐R2 (5′‐GCTTTCGCGCTGTTGGAGAG‐3′) [41].

### Prediction of mRNA structure for alkane biosynthesis genes

**Fig. 3:**
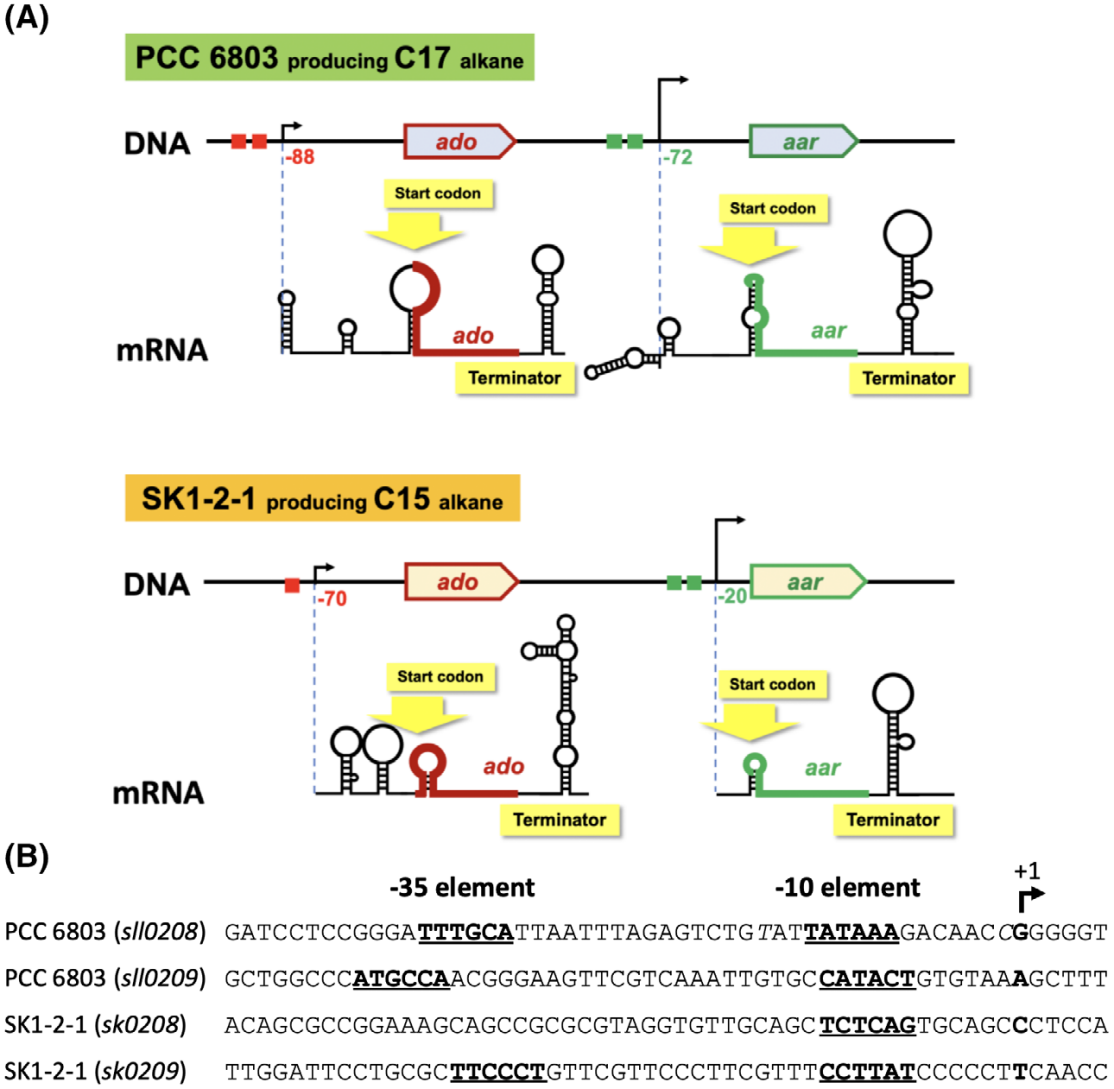
Molecular structures of *ado*/*aar* genes, mRNA and estimated promoters. (A) Calculation and prediction of mRNA structures were performed using the software GENETYX‐MAC ver.15.0.5. The mRNA contains stem‐loop structures at 5′‐ and 3′‐UTR, respectively. Positions of the structural genes are indicated by *ado* (red) or *aar* (green). (B) Consensus promoter sequences are indicated in bold and are underlined. +1, TSS.

Calculation and prediction of mRNA structures were performed using the Genetyx software (GENETYX‐MAC ver. 15.0.5: NIHON SERVER Co. Ltd., Tokyo, Japan), with the following settings: minimum length of stacking region, 4 or 3; maximum stacking energy, −40 or −20 kcal·mol^−1^; suboptimal range, 5%; folding distance, 5 (Fig. 3; Figs S2 and S3). The value of ΔG was predicted by a minimal setting range within 5 bp for a S&L structure (Figs S2 and S3).

### Prediction of 3D structure for ADO/AAR enzyme

3D structures of the proteins were predicted using AlphaFold2 (ColabFold v1.5.5: DeepMind Co., Ltd., Mountain View, CA, USA). Proteins were visualised using the pymol v2.5.0 software (Schrödinger Co. Ltd., New York, NY, USA) (Fig. S7).

### Alkane measurements

**Fig. 4:**
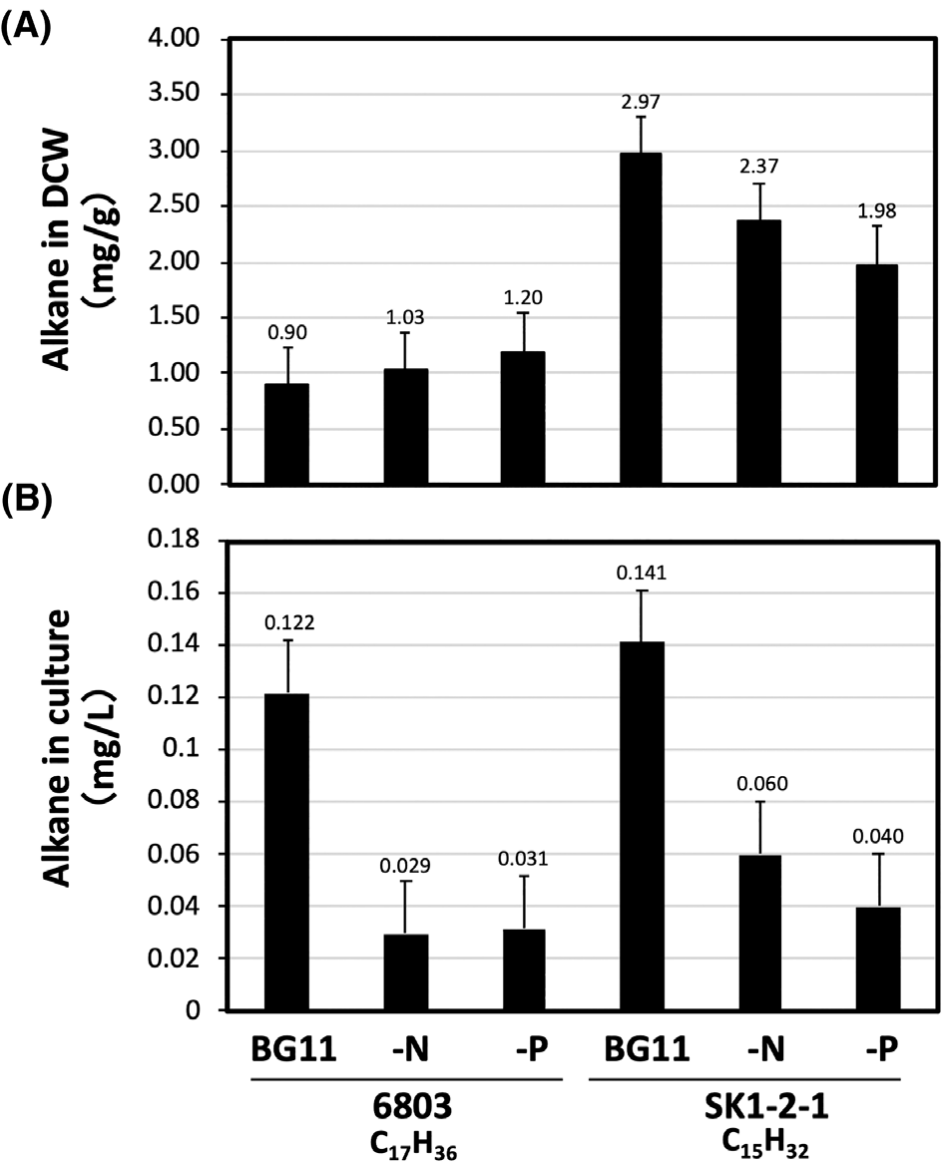
Alkane production depending on culture conditions. PCC 6803GT and SK1‐2‐1 cells were cultivated under 2% CO_2_‐air supplied to BG11, ‐N, or ‐P medium for 6 days (Materials and Methods), and alkane production was analysed by GC–MS. The results are shown as alkane production in (A) mg/g‐DCW and (B) mg/L‐culture. Error bars represent the standard error (*n* = 3). Eicosane (C_20_H_42_) was used as an internal standard, and the specific gravity of the alkanes was calculated to be 0.8.

**Fig. 5:**
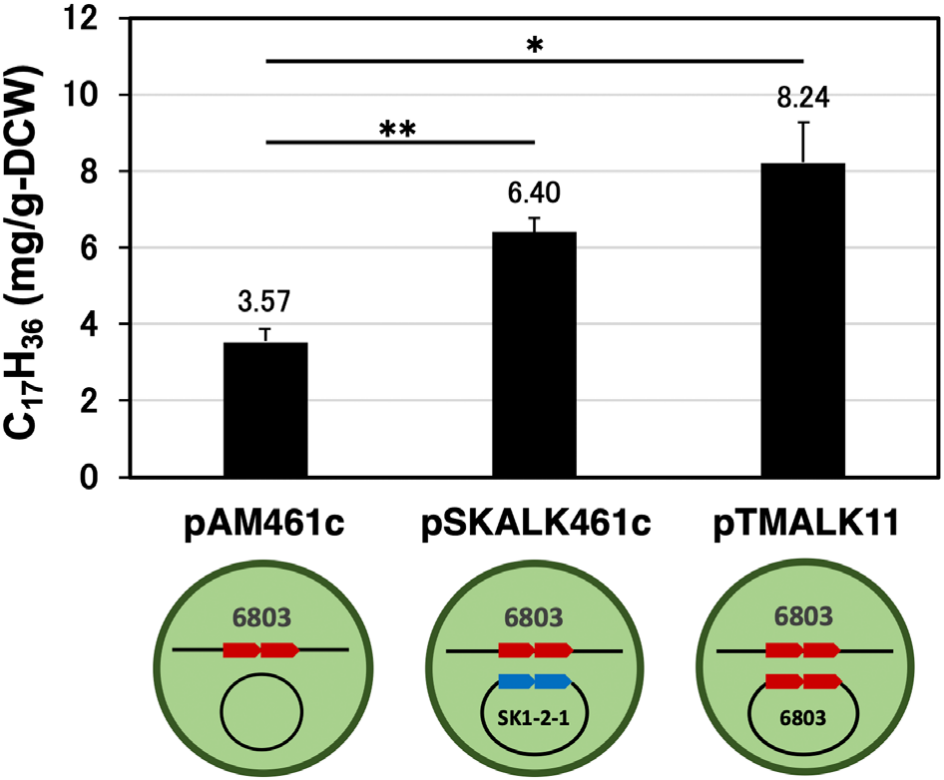
Enhancement of C17‐alkane production in PCC 6803GT_TCs. *Synechocystis* sp. PCC 6803GT transconjugants (TCs) harbouring pAM461c (no insert), pSK‐ALK461c (SK1‐2‐1 *ado*/*aar*) or pTM‐ALK11 (PCC 6803 *ado*/*aar*) were cultivated in BG11 medium exposing to air (0.04% CO_2_) for 4 weeks. After incubation, alkane accumulation was analysed using GC–MS and is expressed as mg/g‐DCW. Error bars represent the standard error (*n* = 3). *P*‐values for all treatment groups, except for the control (pAM461c) group. Eicosane (C_20_H_42_) was used as the internal standard, and the specific gravity of the alkanes was calculated to be 0.8. *t*‐test was also performed, which resulted in significant values for *P < 0.05; **P < 0.01.

Sample preparation and GC–MS analysis (Figs 4 and 5; Tables S2 and S3, Figs S4 and S5) were performed in order to measure the alkanes (hydrocarbons). Freeze‐dried cyanobacterial cells were extracted by sonication with 2 mL of extraction solution [chloroform: methanol = 2 : 1, (v/v)] for 10 min, and the extract was left (static conditions) at −30 °C for 1 h. Prior to sonication, 2 μL of a stock solution of eicosane (C_20_H_42_, 10 000 ppm) was added to the cell lysate as an internal standard for alkane analysis. The supernatant was collected after centrifugation (10 °C, 8000 ***g***, 10 min) and transferred into a 2 mL microtube. In order to wash the extracted solution, the resultant solution (approximately 1 μL) was added to an equal volume of sodium chloride solution [5%, (w/v)] and mixed well. The upper phase was collected after the separation under static conditions. Washing was performed thrice. The resultant solution (approximately 1 μL) was concentrated and dried by an evaporator. The dried samples were then dissolved in 1 mL of n‐hexane, in which eicosane concentration was 20 ppm. An aliquot (1 μL) of the solution was subjected to GC–MS analysis [31]. The analysis was performed using a 6890 N Network GC system (Agilent Technologies Co. Ltd., Santa Clara, CA, USA) and a JEOL JMS‐GC mate II/B GC–MS system. Helium (1 mL·min^−1^) was used as the capillary gas, the injector temperature was 250 °C, and the temperature of a column Zebron ZB‐5MS (7HG‐G010‐11: Phenomenex Co. Ltd., Torrance, CA, USA) was initially kept at 100 °C for 1 min, then increased by 5 °C in 1 min increments to 150 °C. The temperature was then raised in 5 °C increments of 5 °C per minute to 150 °C and from there raised in 10 °C increments of 10 °C per minute to 250 °C. The temperature was maintained for 15 min. During injection, a syringe was used to aspirate 0.3 μL of air, 1 μL of sample, 1 μL of air and 1 μL of acetone, in that order.

### Microscopic observations

**Fig. 6:**
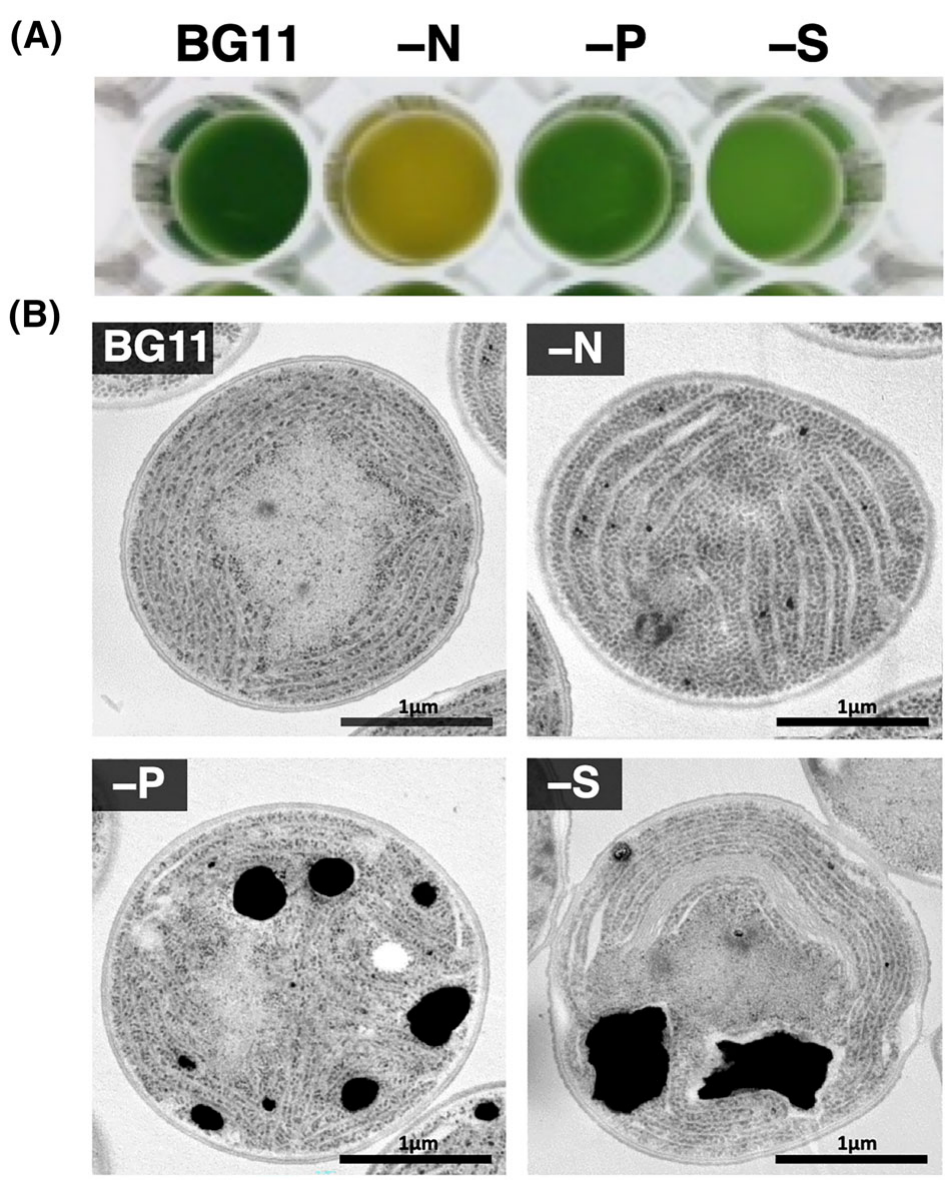
Observations of the PCC 6803GT transconjugant (TC) cultivated under nutrient‐depleted conditions in BG11 medium. (A) Cell culture. TCs harbouring pTM‐ALK11 (PTM) of PCC 6803GT were precultivated in BG11 medium for 4 weeks. Twenty millilitres of the cell culture was collected by centrifugation, and the cells were suspended in 20 mL of nutrition‐depleted BG11 medium: BG11 without NaNO_3_ (nitrogen depletion, ‐N), BG11 without K_2_HPO_4_ (phosphorus depletion, ‐P), or BG11 without MgSO_4_·7H_2_O (sulphur depletion, ‐S), in a 200‐mL Erlenmeyer flask. The flask was exposed to air (0.04% CO_2_) in a cultivation chamber and subjected to continuous reciprocating shaking (50 rpm) under LL (continuous white light) conditions. After 6 days of cultivation, a portion of the cell culture was collected from the medium and dispensed into a 96‐well plate. (B) TEM electron microscopy. The samples shown in Panel A were subjected to transmission electron microscopy TEM (×18 400) using an ultrathin sectioning method (Materials and Methods, Fig. S6). The bar indicates 1 μm.

The cells were morphologically observed using an optical photomicroscope (BX53/DP72; Olympus, Tokyo, Japan) at high resolution with a Nomarski prism (optics, differential interference contrast) [30, 31, 45]. For TEM observations, 20 mL of each cell culture from different nutrient‐depleted BG11 (‐N, ‐P, ‐S) media was mixed with 10 mL of the cell culture from BG11 medium (volume ratio = 2 : 1, Fig. S6). The cell mixture was fixed with the reagents, 2% formaldehyde (PFA) + 2% glutaraldehyde (GA) in 0.05 m cacodylate buffer (pH 7.2) for prefixation, and 2% osmium tetraoxide in 0.1 m cacodylate buffer for postfixation. The resulting cells were stained with uranyl acetate and lead solution and observed using TEM (JEM‐1200EX: Jeol Co. Ltd., Tokyo, Japan) with an ultrathin sectioning method [46, 47] (Fig. 6).

### Statistical analysis

Statistical analysis of the data was conducted using Student's *t‐*test. The analysis software used was R (www.r‐project.org).

## Results and discussion

### Alkane biosynthesis in cyanobacteria

As shown in Fig. 1, the enzymes responsible for the synthesis of C15‐ or C17‐alkane in cyanobacteria are ADO and AAR. To date, previous studies on the increased alkane synthesis in cyanobacteria have mainly focused on the catalytic activities of enzymes or recombinant methods. In this study, we focused on the structure and expression of biosynthesis genes in the unicellular *Synechocystis* sp. PCC 6803 (*ado*, *sll0208*; *aar*, *sll0209*) and filamentous *Limnothrix* sp. SK1‐2‐1 (*ado*, *sk0208*; *aar*, *sk0209*) cells produce C17‐ and C15‐alkane, respectively. The catalytic activities of the alkane enzymes are discussed based on the results of analyses using recombinant strains. A method for efficiently increasing alkane production with the target carbon chain length when fatty acyl‐ACP is used as a substrate in cells is also discussed.

### Gene structure and transcript expression for alkane biosynthesis

The transcription start sites and transcript levels of the alkane biosynthesis genes *ado*/*aar* were analysed using the primer extension method in two distinct cyanobacteria, PCC 6803 and SK1‐2‐1 (Fig. 2). For transcript analysis, it was extracted from the cell state after one day of relatively short incubation (Fig. S1A,B), rather than from a state where a plateau had been reached after a long incubation. This was because we thought that this would allow us to observe the changes in transcript expression depending on the cultivation conditions more clearly. The template DNAs used to generate sequencing ladder during primer extension analysis are shown in Fig. S1C. First, we determined the Blue Green‐11 medium (BG11, ‐N, ‐P)‐dependent gene expression and transcription start sites (5′‐end mapping of transcripts) of *ado* and *aar* genes (Fig. 2, top) using the PCC 6803 or SK1‐2‐1 strain producing C17‐ or C15‐alkane, respectively. The results obtained using the methods described in the Materials and Methods section are shown in Fig. 2 (middle). The accumulation of mRNA transcribed from the transcription start site of the gene was evaluated by quantifying the band signal intensity on X‐ray film (Fig. 2, bottom and Table S1). When the transcription start site (+1 as 5′‐end of a transcript) is set from A (adenine) of a start codon AUG, a signal was confirmed at −88 or −72 upstream on *ado* and *aar* gene in PCC 6803 cells, respectively (Fig. 2A). According to the reports of RNA‐seq analysis by Kopf et al. [48], the transcription start site of *ado* and *aar* gene was −89 and −72, respectively. The transcripts tended to be induced particularly under nitrogen‐depleted BG11 culture (BG11‐N) conditions within one day. Next, *ado* and *aar* mRNA analysis was also performed in SK1‐2‐1 cells. A signal as transcription start point (+1) was confirmed at −70 and −20 of *ado* and *aar* gene, respectively (Fig. 2B). The relative amount of the transcripts might also be more significant in BG11‐N than those of BG11‐P and/or BG11 culture condition. These results indicate that the accumulation of both *ado*/*aar* mRNAs was effective within one day under nitrogen‐depleted conditions in the cells of the two representative cyanobacteria. However, as discussed in a later section (Fig. 4), nitrogen‐depleted culture conditions are not always optimal for alkane production.

In contrast, RNA‐seq analysis of *ado* and *aar* mRNAs in PCC 6803 cells cultured in BG11 medium revealed an approximately 7‐fold higher transcript accumulation of *aar* than that of *ado* [48]. It was difficult to definitively conclude that the relative amount of transcripts of both PCC 6803 and SK1‐2‐1 *ado*/*aar* in the unit total RNAs might have been greater *aar* than *ado* from the present analysis. We considered that if the sequence ladder concentrations of *ado*/*aar* could be made the same (by further increasing the efficiency of the DNA polymerase synthesis reaction using the dideoxy method in *aar*), it would be possible to compare the *ado* and *aar* mRNA amounts. Therefore, the template DNA changed from a linear form to a super coil (Fig. S1C), and the primer extension was repeatedly performed. However, the results were the same as those shown in Fig. 2 (data not shown). The transcript signal and sequence ladder of the *aar* genes in both PCC 6803 and SK1‐2‐1 may depend on the difficulty of cDNA synthesis by reverse transcriptase and DNA polymerase in response to the structures of mRNA and template ssDNA of the *aar* gene.

### Estimated mRNA structure and promoter sequence

Based on the results of 5′‐end mapping for the transcript, secondary structures at 5′‐ and 3′‐untranslated reader region (UTR) and promoter sequences were estimated for the genes. The molecular structures of the two cyanobacterial species were compared to determine similarities. A summary of the results is presented in Fig. 3. First, PCC 6803 *ado* mRNA showed three stem and loop (S&L) structures on 5′‐UTR and a ρ‐factor independent terminator (Gibbs free energy ΔG = −13.8 kcal·mol^−1^, Fig. S2A) on 3′‐UTR (Fig. 3A). In particular, the upstream S&L of 5′‐UTR may have an effect to improve mRNA stability by inhibiting degradation from 5′ → 3′ exoribonuclease [49]. In contrast, an AU‐rich box (UUUUAA) may present adjacent to the upstream ribosome‐binding sequence (AGGAG). The AU‐rich box is a possible target site for RNase E/G‐type endoribonuclease cleavage, conferring mRNA instability in global regulation [38, 50, 51]. Interestingly, the ribosome‐binding sequence and initiation codon (AUG) are present in the third S&L structure. In general, such structures are known to affect the translation initiation [52, 53]. Second, PCC 6803 *aar* mRNA also showed three possible S&L structures in 5′‐UTR and a ρ‐factor independent terminator (ΔG = −10.29 kcal·mol^−1^, Fig. S2B) in 3′‐UTR (Fig. 3A). In this case, there may be an AU‐rich box (UAUUUAUU) at 5′‐UTR on the adjacent upstream of a ribosome‐binding sequence (AGCUA). Furthermore, the ribosome‐binding sequence and initiation codon (AUG) were present in the third S&L structure, similar to the structure in PCC 6803_*ado*. Third, SK1‐2‐1 *ado* mRNA also involve three S&L structures on 5′‐UTR and a terminator (ΔG = −29.0 kcal·mol^−1^, Fig. S3A) on 3′‐UTR (Fig. 3A). This structure was similar to the PCC 6803 *ado* transcript. A possible ribosome‐binding sequence (GGAGAA) and start codon (AUG) were also located in the S&L structures. Fourth, SK1‐2‐1 *aar* mRNA possessed one S&L structure on 5′‐UTR and a terminator (ΔG = −20.82 kcal·mol^−1^, Fig. S3B) on 3′‐UTR (Fig. 3A). Therefore, the study is the first to suggest partially similar mRNA secondary structures with *cis*‐elements on 5′‐ and 3′‐UTR of alkane biosynthesis genes in cyanobacteria. This indicates that similar gene regulation of mRNA stability and translation initiation for alkane biosynthesis may occur in cyanobacteria. Further analysis may be needed to determine whether mRNA stability dependent on 5′‐UTR structure is responsible for higher transcript accumulation of *aar* than *ado* in both PCC 6803 and SK1‐2‐1. Furthermore, it would be interesting to explore whether the mRNA levels are increased directly by transcription level or a stability change at mRNA 5′‐UTR under nitrogen‐depletion conditions.

Possible promoter sequences were estimated from the selected transcription start site (TSS) and are summarised in Fig. 3B. Some variations in similarities, such as consensus sequences at −35 (TTGACA) or −10 (TATAAT) recognised by group 1‐ and/or group 2‐type sigma factor of RNA polymerase, were observed among the four alkane biosynthesis genes. In PCC 6803, *ado* and *aar* are monocistrons expressed in distinct transcript units [54]. Therefore, *ado* and *aar* transcription in cyanobacteria can be independently driven by RNA polymerase [39]. Based on the low similarities in promoter sequences between the two alkane biosynthesis genes of PCC 6803 and SK1‐2‐1, the tendency for significant transcript accumulation under BG11‐N medium conditions in this study may depend on the regulation of mRNA stability rather than transcription efficiency.

### Alkane production depending on culture conditions

Alkane accumulation was measured in the cells grown in BG11, BG11‐N and BG11‐P, and the results are shown in Fig. 4, Table S2 and Fig. S4. In PCC 6803 cells, although C17‐alkane production slightly increased per gram of dried cell weight (g‐DCW) under BG11‐N and‐P culture condition (Fig. 4A), BG11 cultivation condition was significantly more efficient for alkane production per litre culture than that of BG11‐N and‐P (Fig. 4B). The results showed that BG11‐N and BG11‐P tended to increase the amount of alkanes in DCW, but the biomass was found to be much less than that of BG11. Therefore, the amount of alkanes in the DCW was lower when PCC 6803 was cultured in BG11, whereas the amount of alkanes in the unit culture medium was higher. This clearly indicates that alkane production may not always be correlated with transcript accumulation in PCC 6803 and/or other cyanobacterial species. In other words, the availability of biomass is important for alkane production, along with efficient expression of the mRNA required to biosynthesise alkanes. (Fig. 2). BG11 culture conditions were found to be the best for C15‐alkane production, both per g‐DCW (Fig. 4A) and per litre (Fig. 4B) in SK1‐2‐1 cells. This result is consistent with previous observations [18].

### Enhanced alkane production by recombinant cells

Alkane production was evaluated in PCC 6803GT transconjugants (TCs) harbouring recombinant expression vectors carrying pAM461c (vector only), pSK‐ALK461c (pAM461c + SK1‐2‐1 *ado*/*aar*) [32] or pTM‐ALK11 (pAM461c + PCC 6803 *ado*/*aar*) (Table 1). Target genes were cloned into expression vectors as derivatives of pAM461c and stably maintained in cyanobacteria [30]. This experimental concept involves the conversion of the C18 fatty acyl‐ACP in PCC 6803 to C15 (or C17) alkanes by ADO/AAR derived from SK1‐2‐1 cells producing C15 alkanes. The results are shown in Fig. 5, Table S3 and Fig. S5. PCC 6803GT TCs harbouring pTM‐ALK11 produced a 2.3‐fold larger amount of alkanes than those harbouring pAM461c, indicating that C17 alkane accumulation was significantly enhanced by C18 fatty acyl‐ACP as a substrate (*P*‐value = 0.01285). In contrast, approximately 1.8‐fold higher amounts of C17 alkanes were synthesised in TCs harbouring pSK‐ALK461c (*P*‐value = 0.00467). Previous studies have shown that *Synechocystis* sp. PCC 6803 only produce C17 alkanes, but not C15 alkanes [10, 31]. In contrast, SK1‐2‐1 produced C15 alkanes but not C17 alkanes [18]. The previous study has been reported that C15‐alkane is not synthesised by alkane synthase using C18 fatty acyl‐ACPs as a substrate [44]. The results of this study suggest that ADO/AAR from SK1‐2‐1 cells can catalyse C18 fatty acyl‐ACP to C17 alkanes but not to C15 alkanes, as in the previous study mentioned above. In conclusion, abundant C15 alkane production requires not only significant ADO/AAR activities but also a large amount of C16 fatty acyl‐ACP as a substrate.

### Observation of PCC 6803 TCs producing alkane

Finally, the transconjugant PCC 6803GT_PTM‐ALK11 strain was subjected to various culture conditions (BG11, BG11‐N, BG11‐P, BG11‐S) to investigate the accumulation of alkanes in the cells, and the results are shown in Fig. 6. The PCC 6803GT strain was used as the host cell in this study because it is expected to enhance alkane production under autotrophic, mixotrophic and heterotrophic [17] culture conditions. When the cells were cultured in nitrogen‐depleted BG11 medium (BG11‐N) for 6 days, the green colour of the cell culture changed to yellow (Fig. 6A). Under the same conditions, the blue‐green colour in phosphorus‐depleted BG11 medium (BG11‐P) slightly faded, whereas that of sulphur‐depleted BG11 medium (BG11‐S) significantly faded. These results indicate that different changes occur in the colour of the culture medium depending on nutrient limitations.

Under the conditions shown in Fig. 6A, the intracellular accumulation of products in PCC 6803GT_PTM was observed in more detail using transmission electron microscopy (TEM), and the results obtained are shown in Fig. 6B and Fig. S6. When the cells were cultivated for 6 days in BG11 medium, spherical cells with apparent thylakoid membranes were observed. On the other hand, when PCC 6803GT_pTM‐ALK11 cells were cultivated in BG11‐N medium for 6 days, sugar granules seemingly filled the cells and the accumulation of some products (lipids and/or carbon materials involving hydrocarbons) was detected along with thylakoid membranes. When the cells were cultivated in BG11‐P medium for 6 days, thylakoid membranes with ambiguous structures (lipids and/or carbon materials involving hydrocarbons) were detected. A high density of large black‐coloured materials, potentially polyphosphates [46], accumulating in cells during phosphorus source depletion to prepare them for starvation, was also observed in these cells. When the cells were cultivated in BG11‐S medium for 6 days, distorted cell shape and accumulation of large‐sized black‐coloured materials, potentially polyphosphates, were observed. Fine particles, possibly sugar granules, were observed. These results indicated that cell shape and accumulated products differed significantly among cells cultivated under nutrition‐depleted nitrogen (‐N), phosphorus (‐P) and sulphur (‐S) conditions. However, it was not possible to determine whether alkanes accumulated anywhere in the cells in a sufficiently noticeable and distinguishable manner. This is clearly different from TAGs that accumulate prominently as oil droplets in cells, such as in green algae [26]. This may be due to alkane productivity (0.1–1% DCW) and/or a specific manner of hydrocarbon accumulation within the recombinant strain. Previous studies reported that alkanes accumulate in the thylakoid membrane space [55]. The present study did not provide sufficient data to support this hypothesis. It is hoped that this will be clarified in the future.

### Gene expression and enzyme activities for enhancing alkane production

**Fig. 7:**
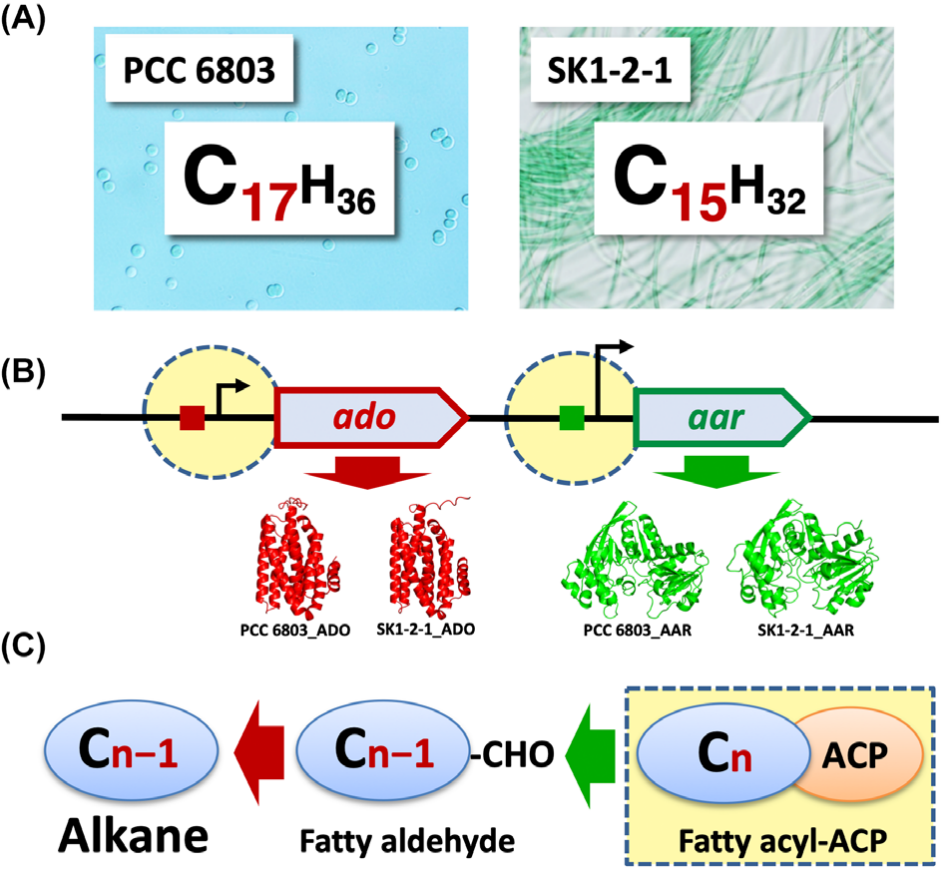
Gene expression and target alkane production in the recombinant cyanobacteria. (A) Cyanobacteria producing C17‐ or C15‐alkane. (B) Gene expression in *ado* and *aar*. (C) ADO/AAR activity. Points to be considered when increasing the alkane production with the desired carbon chain length in recombinant cyanobacteria are indicated by dashed circles or squares.

*Synechocystis* sp. PCC 6803 and *Limnothrix* sp. SK1‐2‐1 are representative cyanobacteria that produce C17‐ and C15‐alkane, respectively (Fig. 7A). Strategies for the efficient production of alkanes with desired carbon chain lengths are discussed below. First, at the transcriptional level, it may be effective to set typical consensus promoter sequences −35 and −10 for the alkane biosynthesis genes. Previous studies have shown that alkane gene expression can be fine‐tuned by altering sequences of promoter and SD (RBS, ribosome‐binding sites) in 5′ UTR [56]. Second, for post‐transcription level regulation, the AU‐box sequence [38, 50], which is a target sequence of ribonuclease, should be eliminated or modified to increase mRNA stability, and the ribosome‐binding sequence and initiation codon should not be hidden within the S&L structure at 5′‐UTR to improve the efficiency of translation initiation (Fig. 7B). At the subsequent enzymatic level of ADO/AAR, amino acid substitutions to improve catalytic activity would be effective [57, 58, 59, 60]. Third, in case of C_*n*−1_‐alkane as the end product, the key will be to produce a large mass of C_n_ fatty acyl‐ACP in the cells. In addition to modifying the underlying lipid metabolism, it is important to increase the number of nontoxic C_n_ fatty acyl‐ACP molecules per cell (Fig. 7C). We hope that the findings of this study can be used to enhance alkane production through recombinant engineering.

## Conclusion

The transcription start sites of the alkane biosynthesis genes *ado* and *aar* were identified in the representative cyanobacteria strains *Synechocystis* sp. PCC 6803 and *Limnothrix* sp. SK1‐2‐1, which produce heptadecane and pentadecane, respectively. This characterisation revealed the potential promoters and unique mRNA structures of the *ado* and *aar* genes in these species. Although nutrient‐depleted media are effective for alkane gene mRNA accumulation, alkane accumulation per unit medium volume is higher when BG11 media is used, given the reduced biomass. PCC 6803 transconjugants carrying alkane biosynthesis genes from PCC 6803 or SK1‐2‐1 showed an approximately 1.8‐ to 2.3‐fold increase in heptadecane production compared to the control strain when grown on BG11 cultures without any nutrient depletion. These results suggest that not only the enzymes ADO/AAR but also the intracellular production of fatty acyl‐ACP substrates may be important for the mass production of target alkanes.

## Conflict of interest

The authors declare no conflicts of interest.

## Author contributions

MA designed the study and MN, TO, HF and YK drafted the manuscript. YK conducted the genome sequencing of *Limnothrix* sp. SK1‐2‐1. MA, MN, TO and HF conducted the experiments. GC–MS was performed with technical support from TO. All authors agreed to the publication of this manuscript in its current form.

## Supporting information


**Fig. S1.** Cultures, total RNAs and template DNAs.
**Fig. S2.** The possible mRNA structures of *Synechocystis* sp. PCC 6803 alkane genes.
**Fig. S3.** The possible mRNA structures of *Limnothrix* sp. SK1‐2‐1 alkane genes.
**Fig. S4.** Alkane production depending on culture conditions.
**Fig. S5.** Enhancement of C17‐alkane production in 6803GT_TCs.
**Fig. S6.** TEM observation of the 6803GT transconjugant cultivated under nutrient‐depleted conditions in BG11 medium.
**Fig. S7.** Possible 3D structures of the ADO/AAR enzymes.
**Table S1.** The band signal intensities of the primer extension under the respective culture conditions.
**Table S2.** Alkane accumulation under the different culture conditions.
**Table S3.** Alkane accumulation in recombinant cells.

## Data Availability

The data that support the findings of this study are available from the corresponding author upon reasonable request.
