# Supplementary material for: Alkane biosynthesis gene expression and its increased production in recombinant cyanobacteria
Source: FEBS Open Bio. 2025 Mar 6;15(6):949–62. doi: 10.1002/2211-5463.70009 (PMC12127877; doi:10.1002/2211-5463.70009)
Supplement: Supplementary file 1 — Fig. S1. Cultures, total RNAs and template DNAs. Fig. S2. The possible mRNA structures of Synechocystis sp. PCC 6803 alkane genes. Fig. S3. The possible mRNA structures of Limnothrix sp. SK1‐2‐1 alkane genes. Fig. S4. Alkane production depending on culture conditions. Fig. S5. Enhancement of C17‐alkane production in 6803GT_TCs. Fig. S6. TEM observation of the 6803GT transconjugant cultivated under nutrient‐depleted conditions in BG11 medium. Fig. S7. Possible 3D structures of the ADO/AAR enzymes. Table S1. The band signal intensities of the primer extension under the respective culture conditions. Table S2. Alkane accumulation under the different culture conditions. Table S3. Alkane accumulation in recombinant cells. [file FEB4-15-949-s001.pdf]

FEBS Open Bio

Supporting Information

Alkane biosynthesis gene expression and its increased production in recombinant cyanobacteria

Misato Nagao,[1,2]† Takato Ozaki,[1]† Hirofumi Fukuda,[1] Yu Kanesaki,[3] and Munehiko Asayama[1,2*]

[*]Correspondence: munehiko.asayama.777@vc.ibaraki.ac.jp

[1]College of Agriculture, Ibaraki University, 3-21-1 Ami, Ibaraki 300-0393, Japan
[2] United Graduate School of Agricultural Science, Tokyo University of Agriculture and Technology, 3-5-8 Fuchu, Tokyo 183-8509, Japan
[3]Research Institute of Green Science and Technology, Shizuoka University, 836 Ohia, Suruga-koi, Shizuoka 422-8529, Japan

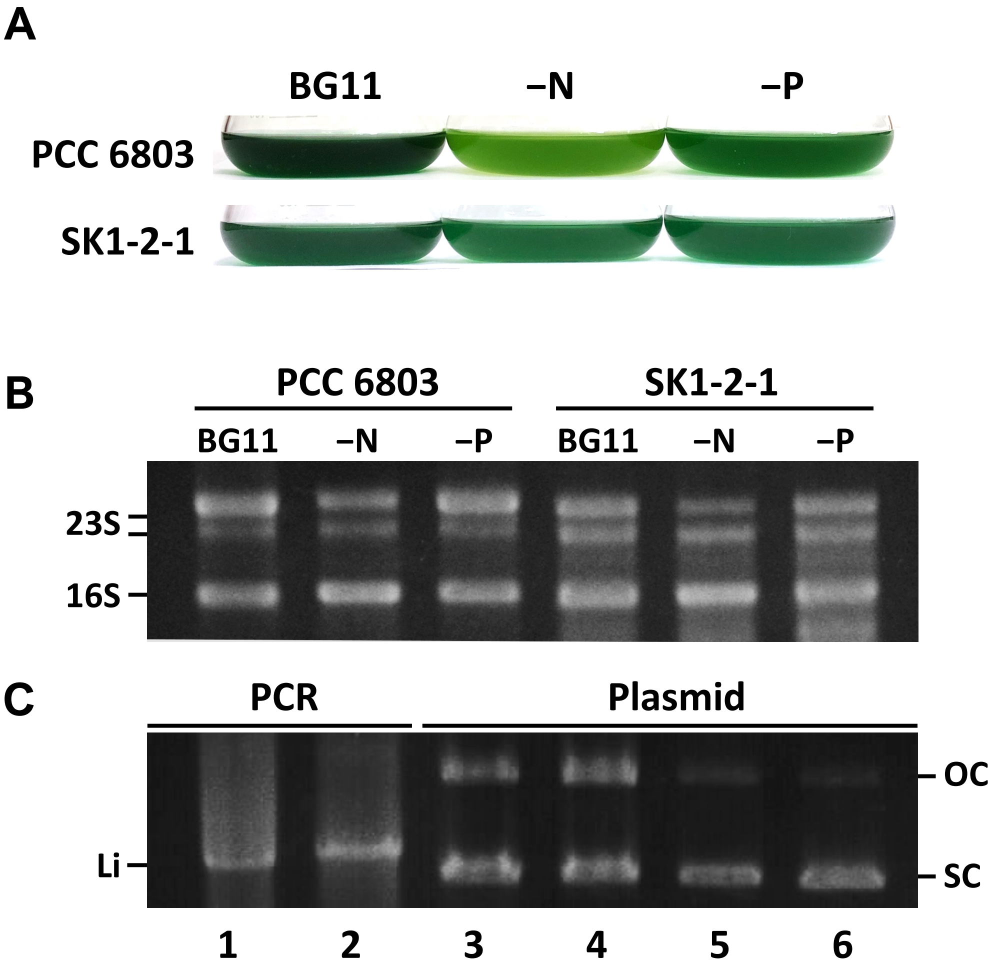

Additional file 1: Fig. S1. Cultures, total RNAs and template DNAs. (A) Cultures of *Synechocystis* sp. PCC 6803GT and *Limnothrix* sp. SK1-2-1 were exposed the nutrient depletion (−N, BG11−N; −P, BG11−P) for 1 day. (B) The photograph shows the extracted total RNAs (2.5 µg each) from the culture indicated in panel A. The positions of 23S and 16S rRNA are shown on a 1.2% agarose gel. (C) PCR-amplified DNAs (PCC 6803, 2.4 kbp: SK1-2-1, 2.5 kbp) or plasmid DNAs (PCC 6803_*ado*, 3.4 kbp; PCC 6803_*aar*, 3.4 kbp; SK1-2-1_*ado*, 3.4 kbp; SK1-2-1_*aar*, 3.3 kbp, Table 1) are shown on a 0.8 % agarose gel. These RNAs and DNAs were used for primer extension analysis as templates for cDNA synthesis and sequencing-ladder synthesis, respectively. 1: PCC 6803_*ado-aar*, 2: SK1-2-1_*ado-aar*, 3: pUCsll0208up, 4: pUCsll0209up, 5: pUCSKsll0208up and 6: pUCSKsll0209up. Li, linear DNA; OC, open circular DNA; SC, supercoiled DNA.

Table S1 The band signal intensities of the primer extension under the respective culture conditions.

6803_ado-88	Signal intensity (%)			
Sample	Exp 1	Exp 2	Exp 3	Average
6803BG11	100	100	100	100 ±0
6803−N	145	165	135	148 ±8.86
6803−P	108	143	121	124 ±10.2

6803_aar-72	Signal intensity (%)			
Sample	Exp 1	Exp 2	Exp 3	Average
6803BG11	100	100	-	100 ±0
6803−N	151	141	-	146 ±4.85
6803−P	130	131	-	130 ±0.34

SK_ado-70	Signal intensity (%)			
Sample	Exp 1	Exp 2	Exp 3	Average
SKBG11	100	100	-	100 ±0
SK−N	123	152	-	137 ±14.4
SK−P	101	118	-	109 ±8.37

SK_aar-20	Signal intensity (%)			
Sample	Exp 1	Exp 2	Exp 3	Average
SKBG11	100	100	100	100 ±0.366
SK−N	135	131	116	127 ±5.93
SK−P	96	98	98	97 ±0.82

Signal intensities as mRNA accumulations from TSS on X-ray films were measured and also shown at the bottom in relative values as % under each cultivation condition (BG11, −N, −P) for 1 day (Fig. 2). Values are presented as mean ± standard error.

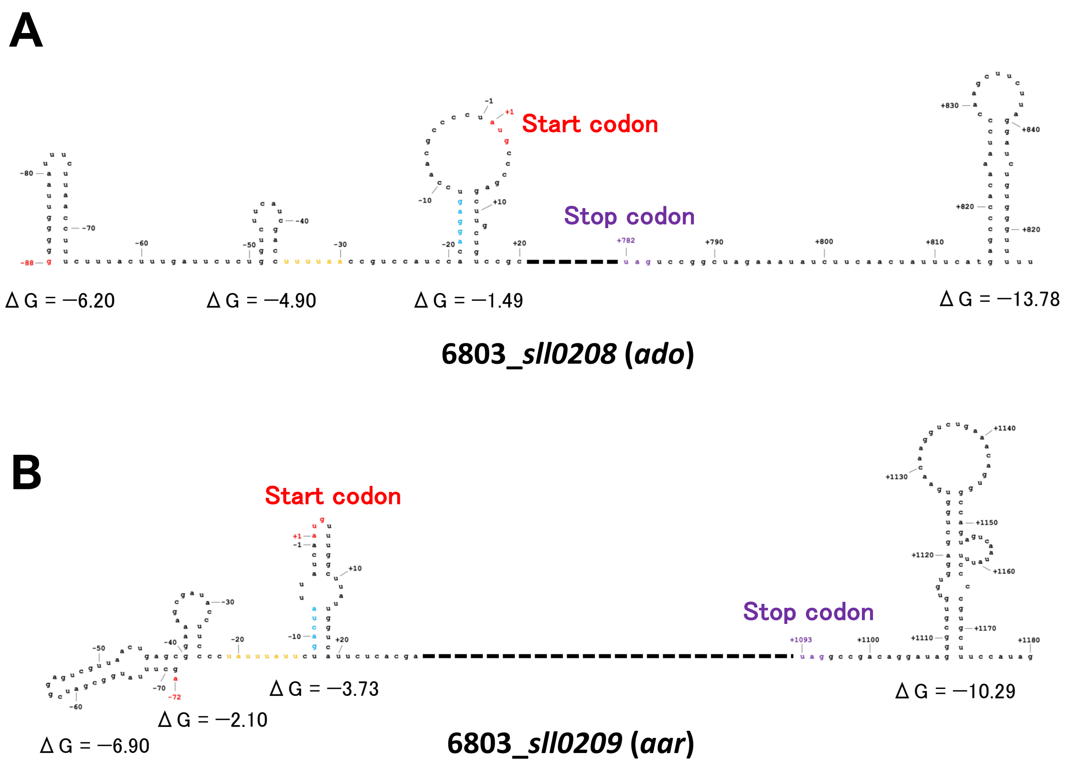

Additional file 2: Fig. S2. The possible mRNA structures of *Synechocystis* sp. PCC 6803 alkane genes. The mRNA secondary structures at the 5'- and 3'-UTR on PCC 6803 *ado* (**A**) and *aar* (**B**) were analysed by a software GENETYX-MAC ver.15.0.5 (NIHON SERVER Co. Ltd., Tokyo, Japan). The transcription start position (red), AU-box (orange), ribosomal binding sequence (blue), start codon (red), stop codon (purple), and stem-loop structure are shown. The values of Gibbs free energy (ΔG, kcal/mol) are also indicated for terminators.

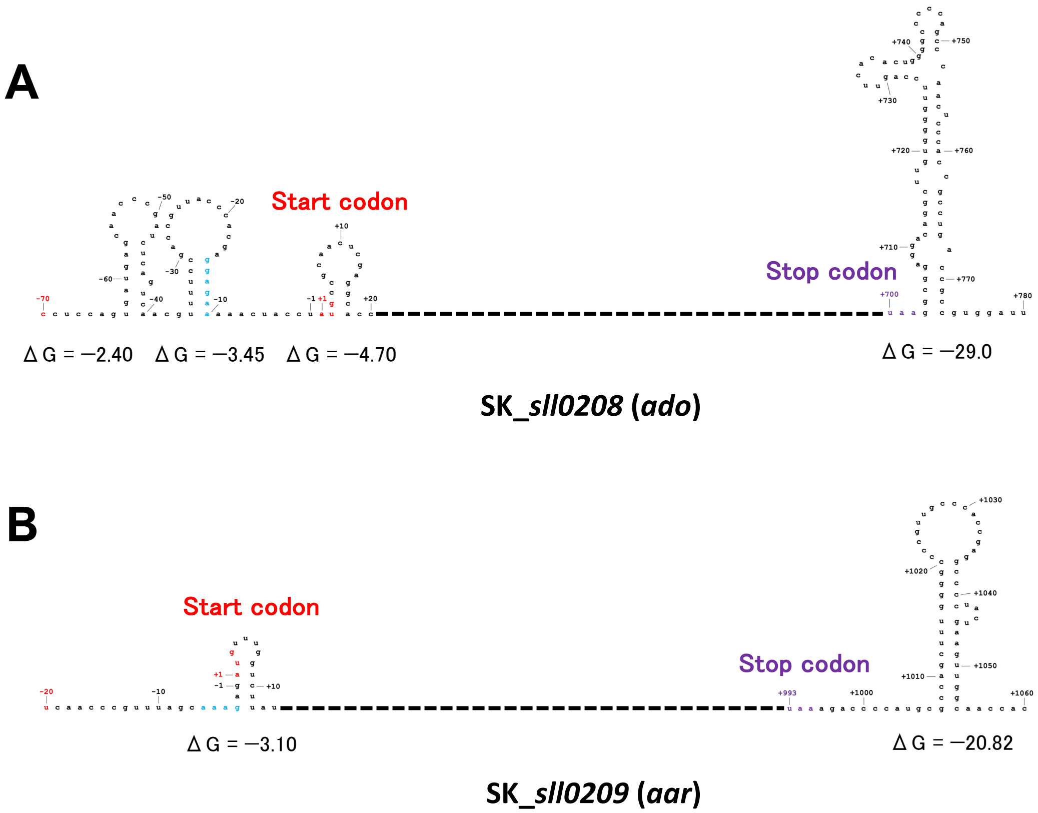

Additional file 3: Fig. S3. The possible mRNA structures of *Limnothrix* sp. SK1-2-1 alkane genes. The mRNA secondary structures at the 5'- and 3'-UTR on SK1-2-1 *ado* (**A**) and *aar* (**B**) were analysed by a software GENETYX-MAC ver. 15.0.5 (NIHON SERVER Co. Ltd., Tokyo, Japan). The transcription start position (red), AU-box (orange), ribosomal binding sequence (blue), start codon (red), stop codon (purple), and stem-loop structure are shown. The values of Gibbs free energy (ΔG, kcal/mol) are also indicated for terminators.

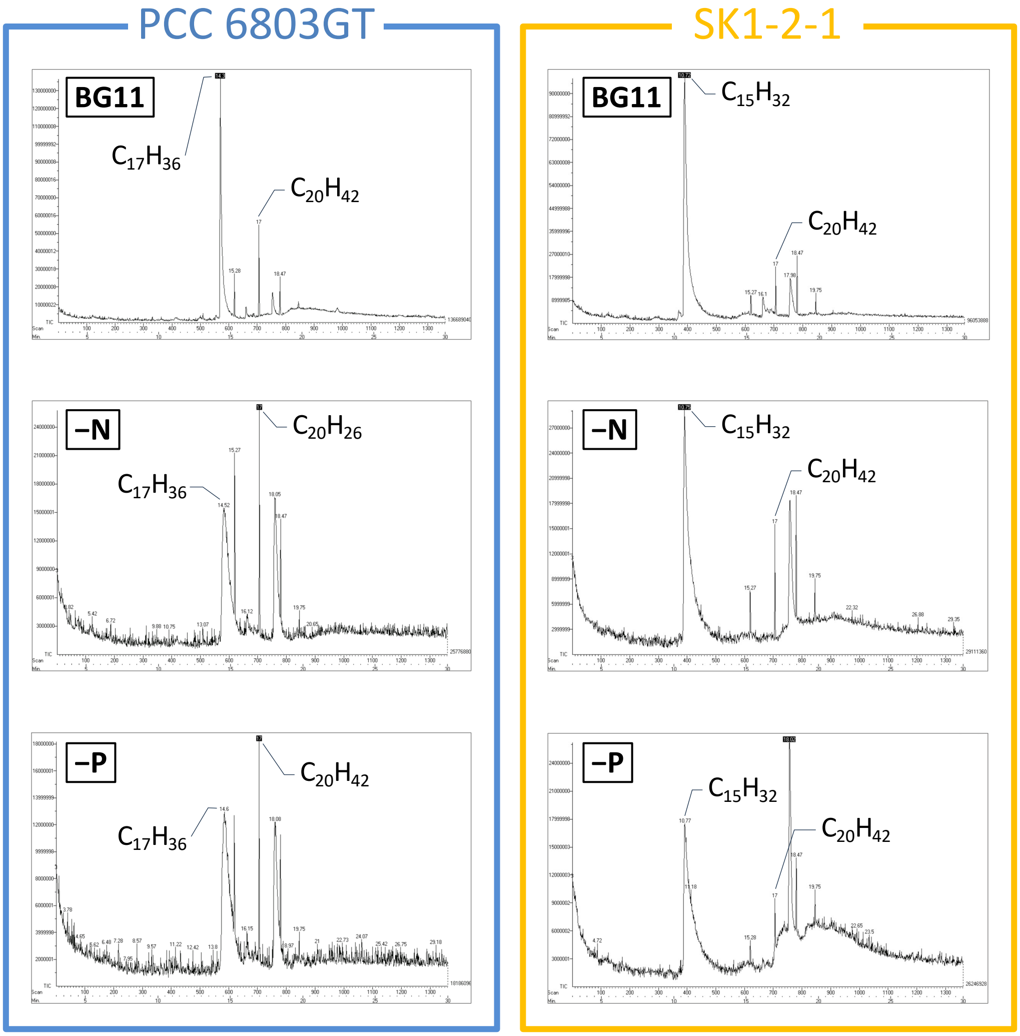

Additional file 4: Fig. S4. Alkane production depending on culture conditions. PCC 6803GT and SK1-2-1 cells were cultivated in BG11, −N, or −P medium supplying 2% CO_2-air for six days (Materials and Methods), and alkane production was analysed by GC-MS. Representative GC-MS profiles of each sample are shown. Eicosane ($C_{20}H_{42}$) was used as an internal standard as a final concentration of 20 ppm. The retention times are 10.7–10.8 min for $C_{15}H_{32}$, 14.3–14.6 min for $C_{17}H_{36}$, and 16.9–17.0 min for $C_{20}H_{42}$, respectively.

Table S2 Alkane accumulation under respective culture conditions.

Sample	Alkane in DCW (mg/g)			
	Exp 1	Exp 2	Exp 3	Average
6803BG11	1.12	1.27	0.96	1.12±0.0907
6803−N	1.59	1.21	1.04	1.28±0.163
6803−P	2.11	1.48	0.90	1.50±0.347
SKBG11	3.68	4.35	3.09	3.71±0.366
SK−N	2.63	2.53	3.73	2.96±0.383
SK−P	2.01	1.36	4.04	2.47±0.808

Sample	Alkane in culture (mg/L)			
	Exp 1	Exp 2	Exp 3	Average
6803BG11	0.00450	0.00571	0.00445	0.00488±0.000411
6803−N	0.00147	0.00111	0.00096	0.00118±0.00015
6803−P	0.00169	0.00119	0.00090	0.00126±0.000228
SKBG11	0.00545	0.00644	0.00506	0.00565±0.000411
SK−N	0.00221	0.00233	0.00268	0.00241±0.000142
SK−P	0.00145	0.00142	0.00194	0.0016±0.00017

Data are presented as mean ± standard error ($n = 3$).

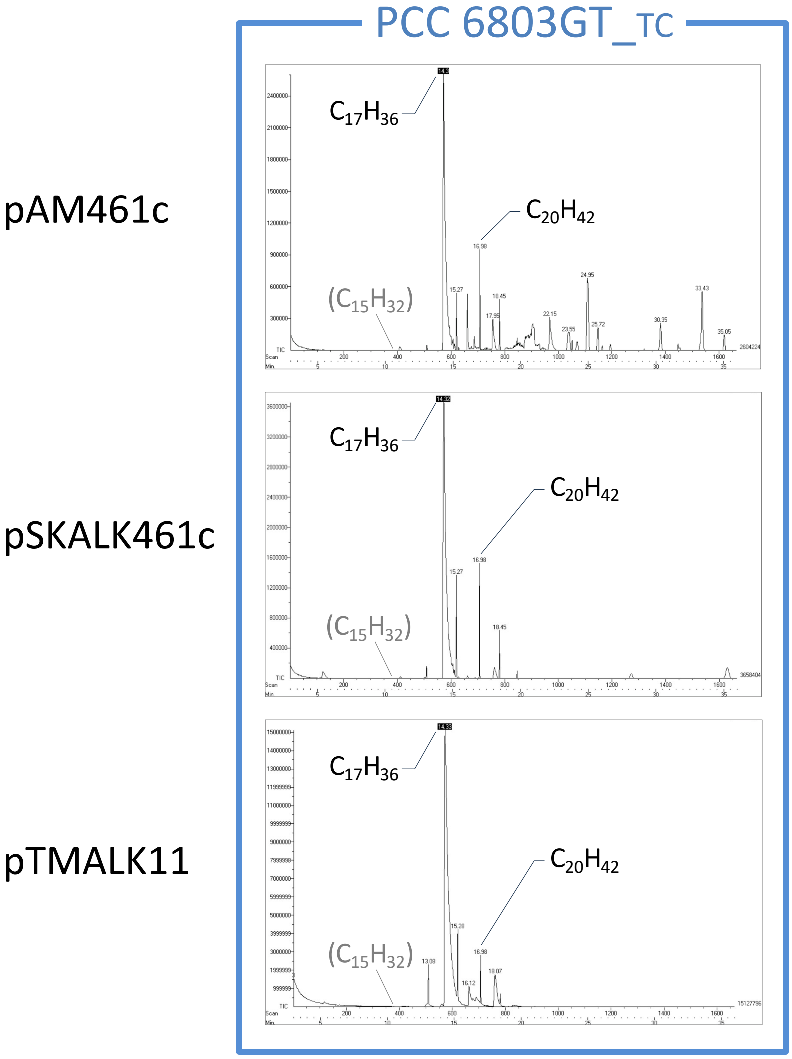

Additional file 5: Fig. S5. Enhancement of C17-alkane production in 6803GT_TCs. *Synechocystis* sp. PCC 6803GT transconjugants (TCs) harbouring pAM461c (no insert), pSK-ALK461c (SK1-2-1 *ado*/*aar*), or pTM-ALK11 (PCC 6803 *ado*/*aar*) were cultivated in BG11 medium exposing to air (0.04% CO_2) for four weeks. After incubation, alkane accumulation was analysed using GC-MS. Representative GC-MS profiles of each sample are shown. Eicosane ($C_{20}H_{42}$) was used as an internal standard as a final concentration of 20 ppm. The retention times are 14.3–14.6 min for $C_{17}H_{36}$ and 16.9–17.0 min for $C_{20}H_{42}$, respectively.

Table S3 Alkane accumulation in recombinant cells.

Sample	$C_{17}H_{36}$ (mg/g-DCW)				
	Exp 1	Exp 2	Exp 3	Average	*p*-value
pAM461c	3.26	4.20	3.24	3.56±0.318	-
pSKALK461c	6.04	7.17	6.00	6.40±0.382	0.00467**
pTMALK11	9.29	6.15	9.28	8.24±1.04	0.01285*

Data are presented as mean ± standard error (n = 3). Values are shown with significant p-values evaluated by t-test as * $p < 0.05$; ** $p < 0.01$.

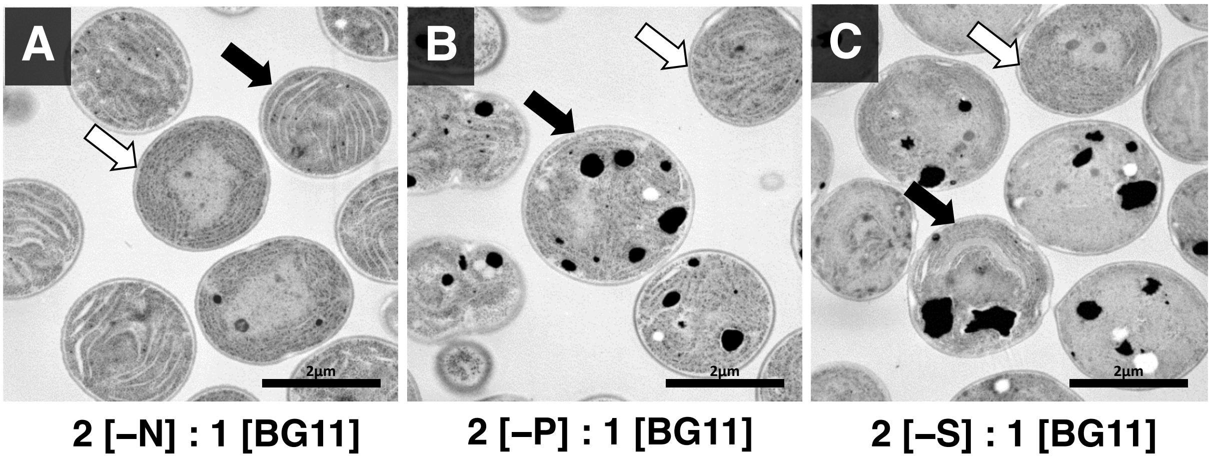

Additional file 6: Fig. S6. TEM observation of the PCC 6803GT transconjugant cultivated under nutrient-depleted conditions in BG11 medium. TC harbouring pTM-ALK11 from PCC 6803GT were cultivated under the conditions described in Fig. 5. After the incubation, two volumes of the respective cell culture grown in nutrient-depleted BG11 were mixed with one volume of the cell culture grown in BG11 media, resulting in a ratio of 2 [BG11 of −N (panel A), −P (panel B), or −S (panel C)] : 1 [BG11]. The cell mixtures were then subjected to TEM analysis. The cells with black arrows represent the respective cells derived from nutrient-depleted BG11 medium, as shown in Fig. 6. Cells with white arrows, derived from the BG11 medium, were also used as controls. The bar indicates 2 µm.

	Synechocystis sp. PCC 6803	*Limnothrix* sp. SK1-2-1
**ADO**	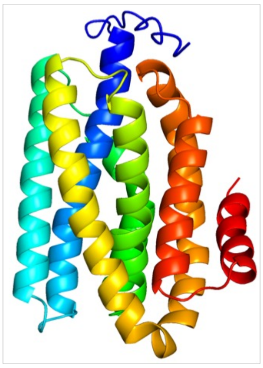	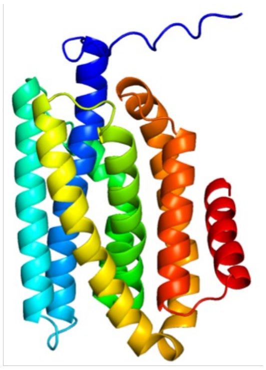
**AAR**	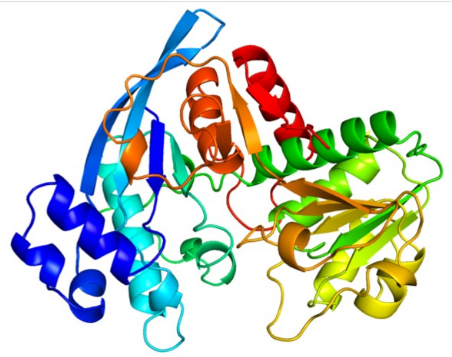	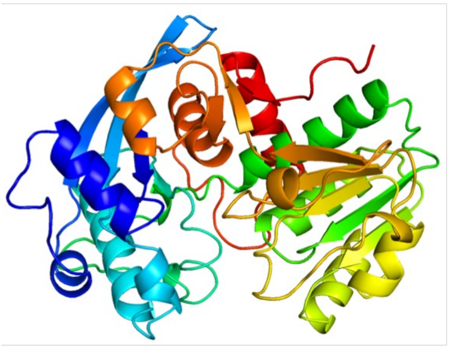

Additional file 7: Fig. S7. Possible 3D structure of the ADO/AAR enzymes. Prediction of 3D structures of ADO/AAR enzymes in *Synechocystis* sp. PCC 6803 and *Limnothrix* sp. SK1-2-1 was performed using the software AlphaFold2 (ColabFold ver1.5.5). Protein visualisation was performed using PyMOL ver.2.5.0. It is colour-coded from blue to red, from the N- to the C-terminus. The amino acid residues are PCC 6803_ADO: 233 aa, PCC 6803_AAR: 231 aa, SK1-2-1_AAR: 343 aa, SK1-2-1_AAR: 340 aa.
